# Surgical approaches to fertility preservation in the cancer patient: current and future directions

**DOI:** 10.3389/fendo.2026.1755050

**Published:** 2026-02-16

**Authors:** Ariella Yazdani, Cara J. Schachter, Elliott G. Richards, Tommaso Falcone

**Affiliations:** 1 Obstetrics and Gynecology Institute, Cleveland Clinic Foundation, Cleveland, OH, United States; 2 Department of Obstetrics and Gynecology, Wake Forest School of Medicine, Winston-Salem, NC, United States

**Keywords:** fertility preservation, oncofertility, ovarian tissue cryopreservation, ovarian transposition, uterine transplant, uterine transposition, trachelectomy

## Abstract

Advances in oncologic therapies have markedly improved survival among reproductive-age female patients, making fertility preservation an essential component of comprehensive cancer care. Gonadotoxic treatments, including chemotherapy, radiotherapy, and surgery, can disrupt ovarian and uterine endocrine function, resulting in premature ovarian insufficiency and infertility. This narrative review summarizes current and emerging surgical approaches to fertility preservation, organized by anatomic focus on the ovary, uterus, and cervix. Ovarian transposition preserves ovarian endocrine function by relocating the ovaries outside the pelvic radiation field, while ovarian tissue cryopreservation preserves primordial follicles through surgical harvesting, cryostorage, and reimplantation to restore ovarian function and fertility. Emerging uterine-preserving strategies, such as uterine transposition and uterine transplantation, demonstrate growing feasibility for restoring reproductive and endocrine uterine function. Fertility-sparing cervical procedures, including radical and simple trachelectomy, maintain favorable oncologic outcomes while preserving reproductive potential in appropriately selected patients. Across all modalities, multidisciplinary collaboration and individualized counseling are critical to optimize both oncologic and reproductive outcomes. Continued research aimed at refining surgical techniques, improve graft viability, and expanding equitable access will be key to advancing fertility preservation as a standard component of comprehensive cancer care.

## 1 Introduction

With new advancements in cancer therapies and improved cure rates, the preservation of reproductive function and fertility has emerged as a vital aspect of long-term survivorship care in reproductive-aged female patients. Cancer treatment regimens are frequently associated with both acute and chronic complications that may compromise reproductive health by impacting both ovarian and uterine function. This paper aims to provide a comprehensive outline of surgical approaches to fertility preservation in the female cancer patient and to highlight research gaps and emerging techniques.

## 2 Methods

A narrative review of the literature was conducted to examine surgical approaches to fertility preservation in reproductive-aged female cancer patients. Relevant articles were identified through searches of academic databases including PubMed and Google Scholar. Search terms included, but were not limited to, fertility preservation techniques (e.g., ovarian transposition, ovarian tissue cryopreservation, uterine transposition, uterine transplantation, trachelectomy) and fertility-related outcomes (e.g., pregnancy, live birth rates, infertility, amenorrhea). Peer-reviewed original studies, review articles, and clinical guidelines relevant to surgical fertility preservation were included; no date restrictions were applied.

The primary outcome of interest was a descriptive synthesis of surgical fertility preservation techniques, with particular attention to reported fertility outcomes. Selected sources were analyzed and synthesized to summarize established techniques, emerging surgical approaches, and gaps in the existing literature. Findings are organized by anatomic focus—ovarian, uterine, and cervical surgeries—and further categorized into current and emerging fertility preservation strategies.

## 3 Background

### 3.1 Impact of chemotherapy treatments on fertility

Standard cancer treatments such as chemotherapy, radiotherapy, and surgery can exert distinct detrimental effects on the gonadal and uterine environment, thereby posing significant risks to future reproductive potential. Because female patients are born with a finite pool of primordial follicles, any injury to the ovary can diminish the ovarian reserve, impair endocrine function, and consequently reduce fertility (1). Fertility potential following cancer therapy is influenced by patient-specific factors, such as reproductive age and baseline ovarian reserve, as well as treatment-related factors such as dosage, treatment class, and the use of combination of modalities like chemotherapy and radiotherapy (2, 3).

Systemic cancer therapy treatment is continuously evolving; however, it is well known that certain chemotherapeutic agents impact ovarian function and thus fertility by varying degrees of toxic effects on ovarian function and follicular reserve known as gonadotoxicity (4–6). The cytotoxic action and subsequent cellular damage imposed by chemotherapeutic agents can result in disrupting cellular growth and ultimately arresting cellular proliferation. In the ovary, this causes a decrease of estrogen, temporarily or permanently causing amenorrhea. In many cases this culminates in a massive destruction of ovarian reserve, known as premature ovarian insufficiency (POI) (7). Along with infertility, a decrease in estrogen further impacts bone function, as well as the cardiovascular and the neurological health of the female patient.

There is strong evidence to support that alkylating and alkylating-like agents cause a significant degree of ovarian damage (4–6). In short, alkylating agents, specifically cyclophosphamide, cause a dose dependent direct destruction of the ova and follicular depletion by causing DNA double-strand breaks leading to apoptosis of primordial follicles resulting in diminished ovarian reserve (1, 8–10). Other potential mechanisms include activation of primordial follicles through the PI3K-PTEN-Akt-FOXO3-mTOR signaling pathway (11–13). Specifically, the “burnout” theory suggests that chemotherapy activates the PI3K-PTEN-Akt pathway, causing follicular loss, reduced AMH-mediated suppression, and subsequent overactivation and depletion of the primordial follicle pool (14, 15). Further, stromal fibrosis and ovarian vascular abnormalities have been described as another mechanism of chemotherapy-induced ovarian dysfunction (16).

Non-alkylating agents, such as doxorubicin have also shown to have a cytotoxic effect on the ovary (17, 18). Doxorubicin inhibits a nuclear enzyme topoisomerase II which causes an accumulation of DNA fragments and stimulates the production of free radicals resulting in mitochondrial damage and ultimately inducing cell death (19). Reproductive damage and thus infertility has been reported in the use of doxorubicin in both childhood and adult patients (20). Moreover, vinca alkaloids (e.g., vincristine), anthracycline antibiotics (e.g., doxorubicin), and platinum-based agents (e.g., cisplatin) have also been shown to impair female reproductive function (7). Despite the already presumed negative effects of certain chemotherapeutic agents on the human ovary, it is difficult to truly examine the gonadotoxicity as many of these agents are not given in isolation and the studies may not accurately examine the full degree of gonadotoxic damage on fertility lifespan as this often requires long-term follow up. Further, there are no pharmacological approaches available to prevent gonadotoxicity in most patients at this time. Gonadotropin-releasing hormone (GnRH) agonists have a limited role for gonadal protection in some cases of breast cancer (21). Additionally, a recent mouse-model study demonstrated that combined treatment with the mTOR inhibitor temsirolimus and recombinant anti-Müllerian hormone (AMH) shows promise as an effective gonadoprotective strategy (22).

### 3.2 Impact of radiotherapy treatments on fertility

In addition to chemotherapy, total body or abdominopelvic ionizing radiation has deleterious effects on gonadal function with the extent of damage influenced by the patient’s age, radiation dose, and treatment field (4, 10, 23). Mathematical models demonstrate the extreme radiosensitivity of the human oocyte, estimating that 50% of human oocytes are destroyed by a radiation dose less than 2 Gy, highlighting the delicate nature of the ovarian reserve (23). Another study developed a predictive model estimating the age at which POI is likely to occur after radiation exposure and introduced the concept of effective sterilizing dose (ESD), or the fractionated dose of radiotherapy (in Gy) at which 97.5% of patients experience immediate ovarian failure. Based on this model, the ESD at birth is 20.3 Gy compared to 14.3 Gy at age 30 years (24). Further, oocyte radiosensitivity varies by developmental stage, with quiescent primordial follicles being more radioresistant than larger, maturing follicles (15, 23).

Radiotherapy not only causes ovarian damage, but it also negatively affects the uterine environment. The high doses of ionizing radiation can cause uterine fibrosis, vascular damage, and hormonal disruption (25). Radiation damages cellular DNA and interferes with normal tissue repair, resulting in the accumulation of collagen and other extracellular matrix proteins. This reduces uterine wall elasticity, potentially limiting the uterus’s ability to expand and support a growing fetus during pregnancy (26). Radiotherapy may also compromise the uterine blood supply, affecting both the endometrium and myometrium. Adequate vascular function is essential for creating a supportive uterine environment, especially during pregnancy, when blood flow must increase to sustain the placenta and developing fetus. Radiation-induced endothelial injury can thicken vessel walls and diminish blood perfusion to uterine tissues, potentially impairing their function (27). Although radiotherapy primarily targets specific tissues, it can also disrupt the hormonal environment necessary for uterine function. Exposure of the ovaries or hypothalamic–pituitary–ovarian axis may reduce estrogen and progesterone, leading to an underdeveloped endometrium, impaired implantation, irregular cycles, and increased infertility or miscarriage risk (28).

### 3.3 Fertility considerations and high-risk cancer types in pediatric and reproductive age oncology

The impact of chemotherapy and radiotherapy on future fertility is a critical consideration in both pediatric oncology and in the treatment of reproductive-age females. Among children, the most common malignancies include leukemia, central nervous system (CNS) tumors, and lymphomas. In premenopausal women, breast cancer (which continues to rise in incidence) represents the most frequent diagnosis, followed by melanoma, cervical cancer, and CNS tumors (29). In particular, there has been a rise in colorectal cancer among young, reproductive-age female patients, highlighting the critical need for surgical interventions aimed at fertility preservation (30, 31). Many of these malignancies are treated with cytotoxic chemotherapeutic agents and/or radiation therapy. Alkylating agents, a class of chemotherapeutics known for their high gonadotoxic potential, are commonly used in the management of leukemia, lymphoma, breast cancer, and multiple myeloma. Similarly, pelvic radiotherapy is frequently employed in the treatment of malignancies involving the gastrointestinal, gynecologic, and urologic systems, where it poses a significant risk to ovarian and uterine function (32). Given these risks, timely implementation of surgical fertility preservation strategies is essential to safeguard reproductive potential in at-risk patients.

## 4 Surgical options for fertility preservation

### 4.1 Indications and patient selection

Fertility preservation should be considered early in the cancer care continuum, ideally at the time of diagnosis. The 2025 American Society of Clinical Oncology (ASCO) guidelines emphasize that all patients of reproductive age should be counseled regarding the potential impact of cancer-directed therapies on fertility, both at diagnosis and during survivorship. Patients expressing interest or uncertainty about future fertility should be promptly referred to a reproductive endocrinologist prior to initiating oncologic treatment (2).

Choosing a fertility preservation method is a complex and nuanced discussion. Patient selection for surgical fertility interventions should consider reproductive age, desire for future fertility, early-stage disease with favorable prognostic features, and scenarios where delaying or modifying standard treatment does not significantly compromise oncologic outcomes. Options include embryo and oocyte cryopreservation, ovarian tissue cryopreservation (OTC), ovarian transposition, and uterine-sparing gynecologic surgeries. Therapy selection may be influenced by patient age, urgency of oncologic therapy, and oncologic diagnosis (2).

### 4.2 Timing of surgical fertility preservation

Surgical fertility preservation can be timed before, during, or after cancer treatment, depending on disease urgency, treatment modality, and patient-specific factors. Ovarian tissue cryopreservation and ovarian and uterine transposition surgeries are generally performed prior to radiotherapy and chemotherapy or at the same time as pelvic surgery. Ovarian and uterine transposition reposition the ovaries and uterus outside the radiation field, but do not mitigate chemotherapy-induced gonadotoxicity. Procedures like radical trachelectomy, or removal of the cervix with preservation of the uterine body, may be performed in select premenopausal women with early-stage cervical cancer. Typically combined with pelvic lymphadenectomy and cervical cerclage, this technique allows for fertility preservation while maintaining oncologic safety.

## 5 Ovarian surgeries for fertility preservation

### 5.1 Ovarian transposition

**Figure 1:**
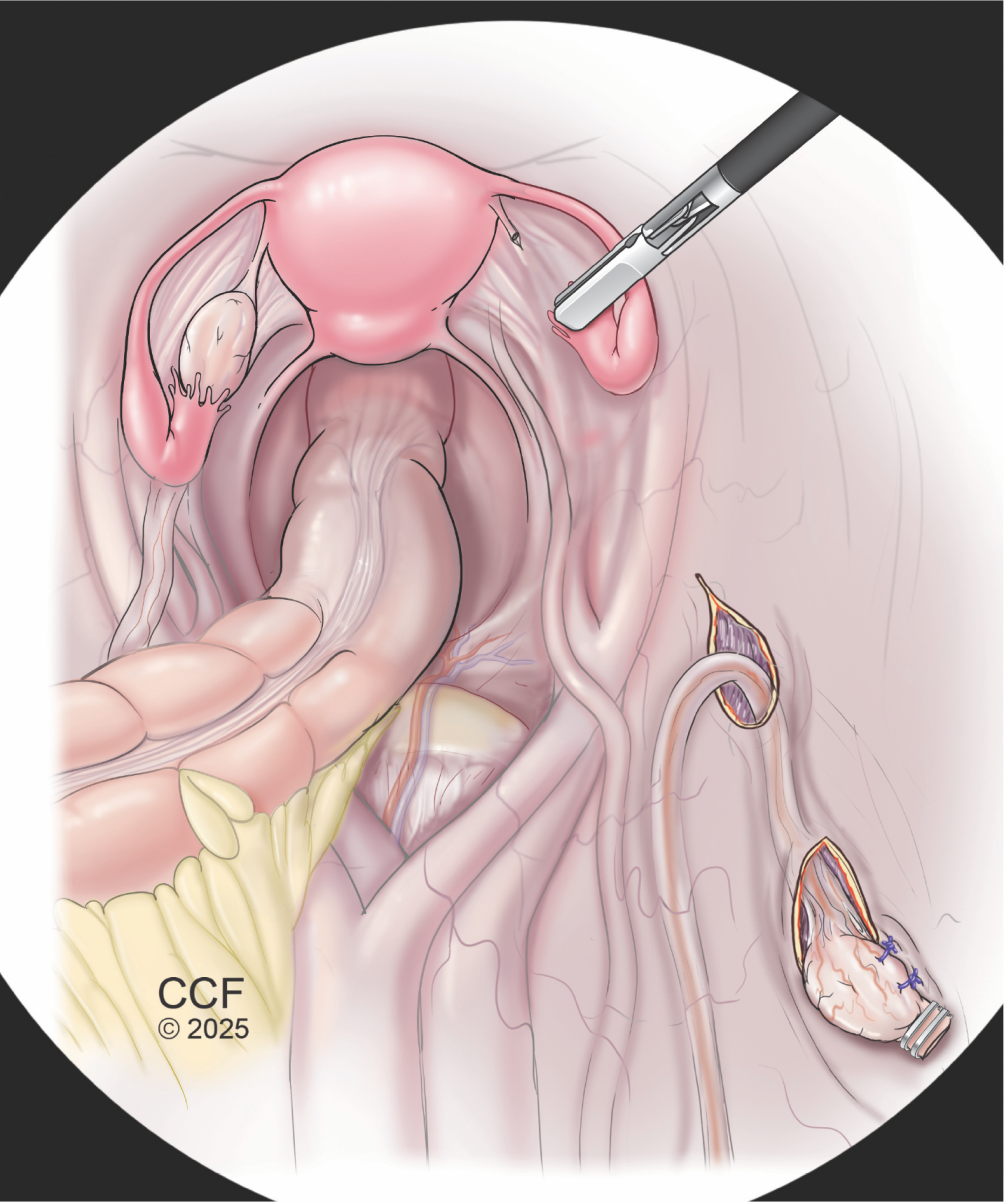
Laparoscopic ovarian transposition for fertility preservation prior to pelvic radiation. Illustration of lateral ovarian transposition, in which the utero-ovarian ligament is transected and the ovary is mobilized on its vascular pedicle within the IP ligament to allow tension-free repositioning cephalad. The ovary is guided through a retroperitoneal tunnel and secured to the ipsilateral anterolateral abdominal wall with non-absorbable sutures to prevent migration. Radiopaque clips may be placed to confirm displacement of the ovary outside the defined radiation field.

Ovarian transposition (OT) is a well-established surgical technique designed to preserve fertility in reproductive-age patients undergoing pelvic or low abdominal radiation therapy (RT). By repositioning the ovaries outside the radiation field, ovarian transposition aims to shield ovarian tissue from ionizing damage that can lead to follicular depletion and ovarian failure (**Figure 1**). Ovarian follicles are understood to be exceedingly radiosensitive; doses as low as 2 Gy have demonstrated up to 50% oocyte damage, and doses in excess of 14 Gy have been shown to precipitate complete ovarian failure (23). Given that standard pelvic RT regimens often exceed 45 Gy, ovarian transposition offers a critical intervention for fertility preservation (4).

Ovarian transposition was first described in a case report of a pediatric patient undergoing radiation therapy for pelvic neuroblastoma (2). The surgical technique behind OT has rapidly evolved over the last six decades. Modern approaches now favor laparoscopic transposition in lieu of laparotomy due to expedited recovery time and earlier initiation of adjuvant therapy. Additionally, radiation oncologists play a pivotal role in delineating the radiation field preoperatively to guide optimal ovarian placement (33).

Ovarian transposition technique varies by individual patient anatomy and oncologic factors. The most common approach is via lateral transposition, which involves transection of the utero-ovarian ligament and mobilizing the vascular pedicle to reposition the ovary superiorly without tension. The primary vascular supply to the ovary, known as the infundibulopelvic (IP) ligament, is carefully mobilized away from the surrounding peritoneum and ureter to allow for complete mobilization of the ovary. A grasper can then be used to develop a retroperitoneal tunnel into which the ovary is guided through. The ovary is subsequently secured to the ipsilateral anterior-lateral abdominal wall using multiple fixation points of non-absorbable suture to minimize tension and migration. Radio-opaque clips may be applied to facilitate intraoperative and postoperative imaging in order to confirm that the ovaries have been moved outside the field of radiation (34). The field for individualized radiation therapy is typically determined pre-operatively and marked accordingly prior to surgery.

Multiple reports comment on the utility of transposing the fallopian tube with the ovary versus leaving the tube in its original position (35). Detachment of the tube may necessitate a more complex discussion about the need for *in-vitro* fertilization for future pregnancy and should be determined on a case-by-case basis. Most often, the fallopian tube is not transected from the uterus and is instead dissected off of the ovary at the level of the mesosalpinx.

Several innovative techniques have been described in regards to ovarian transposition. After initial transposition, ovaries may shift throughout the course of therapy and require repositioning out of the radiation field. Percutaneous needle transposition to the transposed, displaced ovary allows outpatient repositioning post-RT (33). Likewise, few reports describe exteriorization of the ovary into subcutaneous tissue, though this technique carries increased risks of adhesion and hernia formation. Combined approaches, such as ovarian transposition with ovarian tissue cryopreservation, may further enhance fertility outcomes but reliable results are not yet fully understood.

Overall success of ovarian transposition may be influenced by patient age, radiotherapy dose, ovarian distance from the radiation field, and concurrent chemotherapy. A meta-analysis of 29 studies reported preserved ovarian function in 91% of patients undergoing ovarian transposition (36). A separate systematic review of 38 studies found superior ovarian survival with ovarian transposition combined with brachytherapy, which supplies internal localized radiation, compared to external beam radiation therapy, with low complication rates (37). Commonly reported complications of ovarian transposition include ovarian cyst formation (94.9%), abdominal pain (3.4%), and postoperative adhesions leading to small bowel obstruction (0.85%) (38). Although the effect on ovarian function is clear, oncologists and reproductive endocrinologists must weigh the benefits against the risk of incomplete cancer staging, metastatic involvement of transposed ovaries, and port-site metastases. In addition, there exists a separate, albeit rare, risk of unrecognized primary ovarian cancers within the harvested ovarian tissue. For this reason, individualized planning with the multi-disciplinary team, such as pre-operative imaging and intraoperative assessment, should be discussed prior to proceeding with ovarian transposition.

### 5.2 Ovarian tissue cryopreservation and transplantation

**Figure 2:**
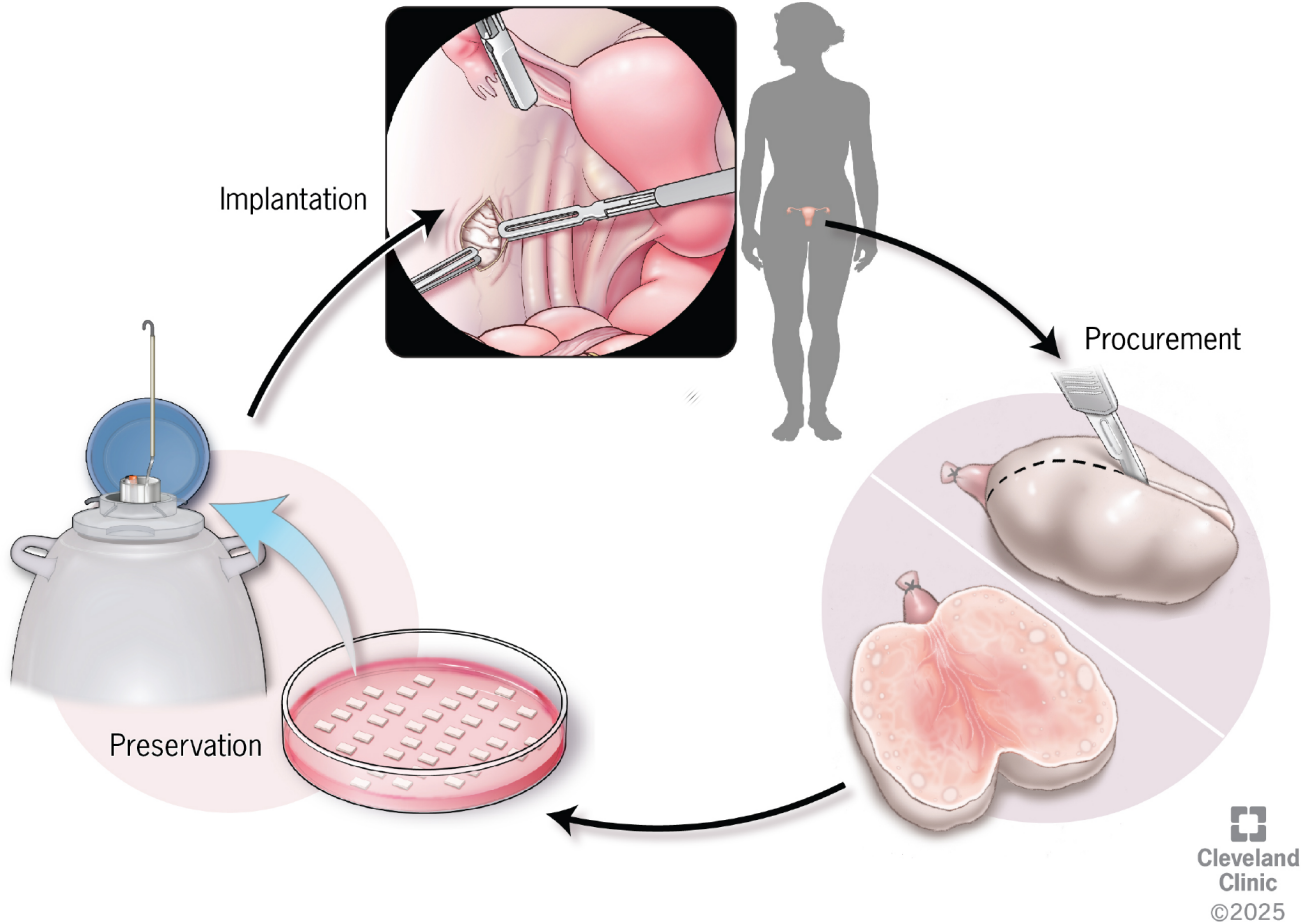
Ovarian tissue cryopreservation and transplantation for fertility preservation. Schematic illustration of the ovarian tissue cryopreservation workflow. Cortical ovarian tissue is surgically procured prior to gonadotoxic therapy and sectioned into thin strips, which are then cryopreserved for long-term storage. When fertility restoration or endocrine recovery is desired, thawed ovarian tissue fragments are transplanted, typically to an orthotopic site within the pelvis, where revascularization allows resumption of ovarian endocrine function and potential follicular development.

Ovarian tissue cryopreservation (OTC) is an important fertility preservation option for female patients at risk of treatment-induced gonadal damage (**Figure 2**). Its primary goal is to preserve primordial ovarian follicles for potential future restoration of ovarian function and fertility. The process involves surgically removing cortical ovarian tissue, followed by cryopreservation and eventual reimplantation after gonadotoxic therapy (39). Unlike other methods, OTC does not require ovarian stimulation or oocyte retrieval, making it the only viable option for prepubertal girls or patients who cannot delay cancer treatment. Once considered experimental, OTC is now increasingly understood as an acceptable fertility preservation technique, however still without standard methods and guidelines on ovarian surgical procurement, tissue processing, cryopreservation, storage, or transplantation technique (40).

Ovarian tissue biopsy should ideally be performed before the initiation of gonadotoxic therapy; however, prior chemotherapy is not an absolute contraindication to OTC. In such cases, ovarian function should be carefully assessed to determine tissue viability and the likelihood of successful preservation (41). For example, patients with an antral follicle count (AFC) of 0-1, an AMH of zero, or post-menopausal FSH levels may derive very limited benefit from OTC and can expect little to no recovery of ovarian function (42, 43). Additionally, the patient age at which OTC is recommended should be considered, as concentration of follicles has been shown to decreased significantly with age (44). Furthermore, majority of reported cases who achieved successful pregnancies were patients in their 20s when ovarian tissue was harvested. Although there is no clear age limit as to when to offer OTC as a fertility preservation option, well-established predictors of ovarian function should strongly factor into both patient selection and counseling.

The surgical approach and the amount of tissue to be removed depend on several factors, including the patient’s age, baseline ovarian reserve, and the risk of iatrogenic POI. For example, in prepubertal girls, unilateral oophorectomy is often recommended due to the small size of the ovaries. Other cases in which oophorectomy may be preferred over biopsy is for patients receiving pelvic radiation or high doses alkylating agents. If biopsy is performed, typically one-half to two-thirds of one ovary or half of both ovaries should be biopsied and cryopreserved (45). Various sites around the world have described ovarian tissue processing into strips, squares or fragments. Although there is no agreed upon processing size or shape of cryopreserved ovarian tissue, a systematic review by Diaz et al. evaluated 58 fertility preservation sites around the world and found that most process ovarian tissues in strips at least 10mm x 5mm (length, width), with a 1-2mm thickness (46). The authors further noted that a 1-2mm thickness has been shown to reduce ice crystal formation during cryopreservation, reduce ischemic time and increase oxygenation once auto-transplanted (46, 47).

No current guidelines or best-practices are outlined for specific ovarian harvest and transplantation techniques. Biopsies are most commonly performed laparoscopically or during planned oncologic surgery via laparotomy. To preserve follicular integrity, the corpus luteum, if present, should be avoided during tissue collection, and energy-based surgical devices should be minimized to prevent thermal damage to the ovarian cortex. Once biopsied, the ovarian tissue should be immediately placed in hypothermic environment to avoid warm ischemia injury. For this reason, very often tissue processing centers are within the same facilities or nearby the site for surgical removal. Cryopreservation methods include both slow freezing or vitrification. The standard method of OTC worldwide is slow freezing which was used in almost all reported pregnancies (41), however more recent studies have shown comparable outcomes with vitrification techniques (48). Ultimately, more high-quality studies are needed to evaluate these two cryopreservation techniques.

Although the concept of OTC has been around for nearly a century, clinical ovarian tissue transplantation (OTT) is rather limited, as the first successful ovarian auto-transplantation of cryopreserved tissue was described in 2000 (49). The first recorded live-birth after auto-transplantation of ovarian cortical tissue was reported in 2004 (50). Ovarian tissue can be transplanted to orthotopic sites, back to the medulla of the ovary or peritoneal pockets in the pelvic cavity, or heterotopic sites, outside of the pelvic cavity, i.e., the forearm or abdominal wall muscle (50). Orthotopic reimplantation, either performed laparoscopically or via mini-laparotomy, is thought to be the most effective in restoring endocrine function and thus fertility (41, 51). Further, orthotopic reimplantation can allow for spontaneous pregnancy in the future, whereas transplantation to a heterotopic site requires future *in-vitro* fertilization (52).

The most common orthotopic transplantation techniques for grafted ovarian tissue are reimplantation into a pelvic peritoneal pocket or directly onto the ovary, ipsilateral, or contralateral (49). Obstetric outcomes do not appear to differ significantly between these two transplantation sites (53). To prepare the transplantation site on the pelvic sidewall or ovary, cold scissors are typically used to minimize injury and preserve vascular supply (54). However, some studies have employed bipolar cautery to achieve improved hemostasis (55). Donnez et al. described a technique involving decortication of the ovary followed by transplantation of the graft. In this method, the ovarian graft is micro-surgically sutured with propylene suture and secured to the medulla of the ovary, after which the graft is covered with oxidized regenerated cellulose (Interceed^®^) or fibrin glue. When this approach is unsuccessful, Donnez et al. proposed placing the ovarian cortical graft within a highly vascular peritoneal pocket on the anterior leaf of the broad ligament, again covering the graft with oxidized regenerated cellulose and fixing the edges using fibrin glue (51). Other reported methods include attaching the graft directly to the peritoneum and subsequently closing the peritoneum with fibrin glue (56). Additional variations have been described that avoid foreign materials altogether, omit peritoneal closure, or use sutures instead of glue to close the peritoneum (55, 57).

There has been a growing number of pregnancies and live births resulting from OTT in recent years (58). It is predicted that over 500 patients worldwide have received OTT, resulting in over 200 live births (58–60). In one systematic review from 2022, Khattak et al. reported pregnancy rates of 37% (95% CI: 28–96%) for frozen, and 52% (95% CI: 28–96%) for fresh transplants, with corresponding live birth rates (LBR) of 28% (95% CI: 24–34%) and 45% (95% CI: 23–86%), respectively (58). Andersen et al. reviewed assisted reproductive technology (ART) outcomes in women with OTT and reported pregnancy rates per cycle of 3.9-19.3% and live birth rates of 3.9-14%, notably lower than in standard IVF populations. They also observed a high incidence of empty follicle syndrome, or a failure to retrieve oocytes despite appropriate follicular development, reported in up to 35% of ART cycles, likely reflecting impaired folliculogenesis in transplanted ovarian tissue. More recently, Li et al. reported that estimated LBRs after cryopreserved OTT vary significantly by grafting site. Transplantation to the remaining ovary showed an LBR of 64%, compared to 31% for transplantation to the pelvic peritoneum. When categorized by transplantation type, the estimated LBRs were 44% for orthotopic, 5% for heterotopic, and 23% for combined orthotopic and heterotopic transplantation (60). Overall, success rates of OTT remain variable and difficult to interpret across studies. The marked variability in reported outcomes underscores the heterogeneity of patient populations, surgical technique, and fertility strategies. Individualized counseling should continue to guide expected success after OTT (61).

#### 5.2.1 Research gaps and emerging directions in OTC

Despite significant advances in OTC and auto-transplantation, several critical research gaps remain that limit their optimization and broader clinical application. A foremost challenge is the substantial loss of primordial follicles following transplantation, largely attributed to ischemic injury during the revascularization phase. Because OTT involves a non-vascularized free graft, the survival and function of the transplanted tissue depend on the restoration of a functional blood supply. The ischemia-induced follicular burnout is driven by disrupted molecular signaling, including altered expression of activator and inhibitory proteins such as GDF-9, BMP-15, KitL, and Tsc1 (62, 63). One systematic review showed that strategies aimed at enhancing neo-angiogenesis (including proangiogenic growth factors, hormonal treatments, antioxidants, anti-apoptotic agents, and adult stem cell therapies) have shown promising initial results, with combination approaches likely offering the greatest efficacy (64).

Studies on neovascularization after OTC and OTT face key limitations. Many rely on xenograft models, typically using immunodeficient mice, which do not fully reflect human immune and vascular dynamics (65–68). Additionally, human studies focus on short-term outcomes, with limited insight into long-term vascular stability or graft function. Variability in cryopreservation methods, graft sites, surgical techniques, and adjunct therapies further complicates comparisons. Critically, early follicle loss due to delayed revascularization remains a major challenge, indicating that while angiogenesis is essential, it alone may not ensure long-term follicle survival or endocrine recovery.

Another pressing concern is the oncologic safety of reimplanting cryopreserved ovarian tissue, particularly in patients with hematologic malignancies (69). Studies have demonstrated that molecular techniques like RT-PCR and xenografting can detect minimal residual disease, revealing the presence of malignant cells undetectable by histology. Strategies to enhance safety in these cases include *in vitro* maturation (IVM), artificial ovarian construction, and stem cell-based oogenesis (64). This underscores the need for robust screening protocols and possibly alternative fertility preservation strategies for high-risk cancer patients.

*In vitro* maturation of oocytes is the maturing of oocytes outside the body to avoid reintroducing malignant cells present in the ovarian tissue on auto-transplantation. It is emerging as a promising avenue, offering fertility preservation without the risks associated with tissue reimplantation (70–73). Although IVM has transitioned from experimental to clinical use, its efficacy and success rates remain suboptimal, necessitating further refinement of culture systems and a deeper understanding of oocyte maturation dynamics. Additionally, the first successful allogenic tissue transplantation with safe immunosuppression has been reported (74). Collectively, these areas highlight the need for multidisciplinary research focused on improving graft viability, ensuring oncologic safety, and advancing ex vivo fertility preservation technologies.

## 6 Uterine surgeries for fertility preservation

While ovarian transposition is a well-established technique to preserve the ovaries from the radiation field, fertility-sparing uterine surgery is currently investigational. Uterine transposition represents an emerging approach aimed at preserving reproductive potential in patients undergoing pelvic radiotherapy, most commonly for malignancies such as cervical cancer, colorectal cancer, or pelvic sarcomas (75). This procedure involves the surgical transposition of the uterus, often in conjunction with the fallopian tubes and ovaries, to a location within the upper abdomen, thereby positioning the reproductive organs outside the planned radiation field. The primary objective is to shield the uterine fundus and adnexa from radiation-induced damage, ultimately maintaining the potential for spontaneous conception and to then carry a pregnancy in selected female cancer survivors.

### 6.1 Uterine transposition

The first uterine transposition surgery was described by Ribeiro et al. in 2017 in a patient requiring pelvic radiotherapy for rectal adenocarcinoma (76). Shortly thereafter, the first recorded live birth was documented in 2022 in a patient who had uterine transposition surgery before undergoing pelvic and thoracic radiation for left iliac and thoracic synchronous myxoid low grade liposarcoma (77).

**Figure 3:**
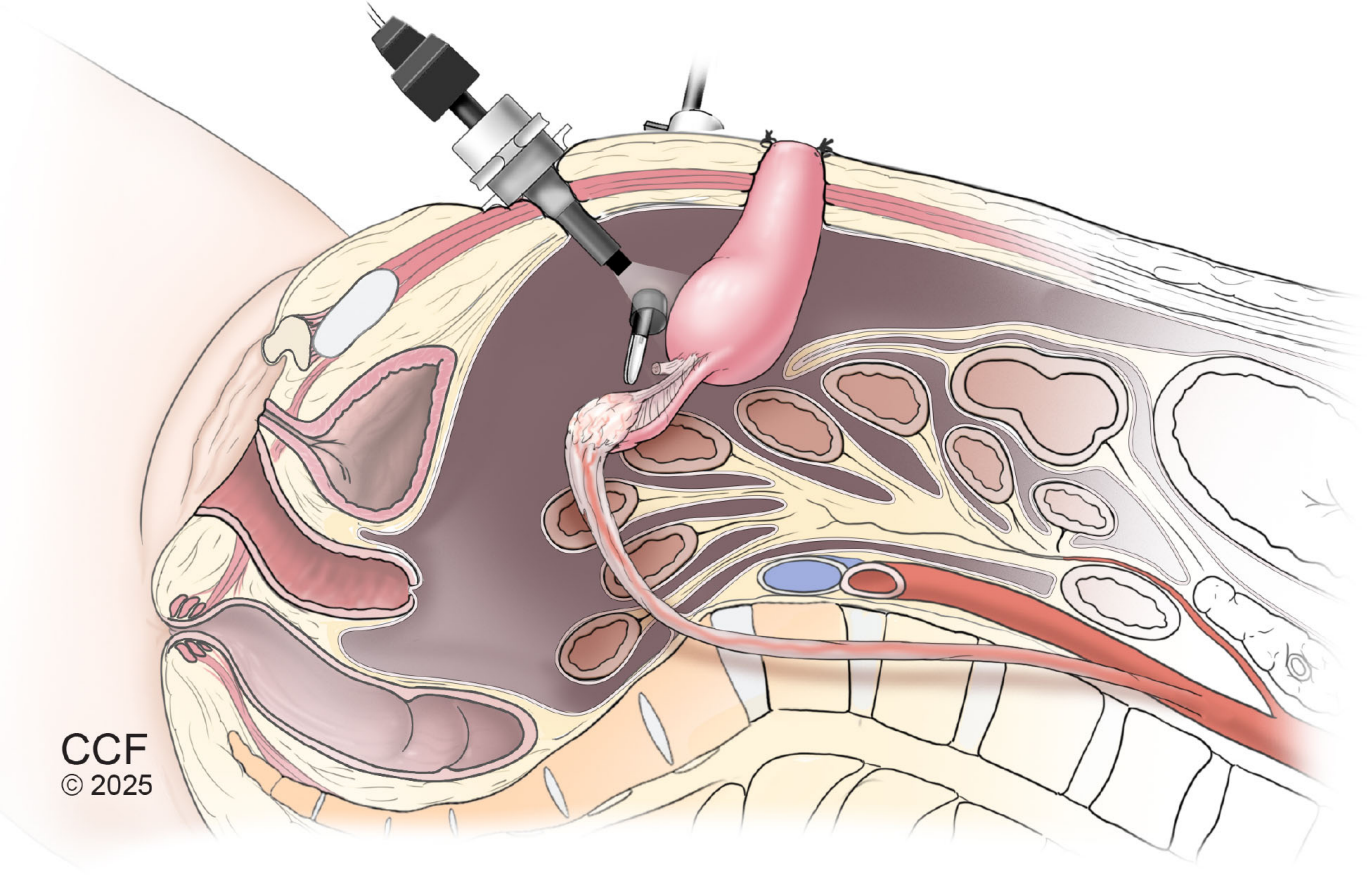
Sagittal view of laparoscopic uterine transposition for fertility and uterine preservation prior to pelvic radiation. Illustration of laparoscopic uterine transposition, demonstrating transection of the round and broad ligaments, ligation of the uterine arteries, and skeletonization and mobilization of the infundibulopelvic ligament with ovarian vessels to enable cephalad displacement. A colpotomy is performed in a manner analogous to total hysterectomy, allowing mobilization of the uterus and adnexa for transposition to the upper abdomen, outside the anticipated radiation field. In select techniques, the cervix may be anastomosed to the umbilicus to create a cervical stoma permitting menstrual outflow and endometrial surveillance, as depicted here.

In both reported cases, a minimally invasive surgical approach was utilized. The procedure involves transection of the round and broad ligaments, followed by skeletonization and mobilization of the IP ligament and its encompassing ovarian vessels to the level of the kidney. The uterine arteries are ligated, and a colpotomy is performed in a manner analogous to that of a total hysterectomy (**Figure 3**). These steps facilitate adequate mobilization of the uterus and adnexa, allowing for their transposition to the upper abdomen, outside the anticipated pelvic radiation field (76, 77). Ribeiro et al. further described the creation of a cervical stoma in which the cervix is anastomosed to the umbilicus, creating a uterine outlet where both menses can resume and evaluation of the endometrium can be performed (75). There are cases in which the cervix remains in the abdomen at the level of the umbilicus with the uterine body fixed above the umbilicus. In these cases, menses is suppressed via medical treatment (77–83).

On average, patients commenced radiation treatment approximately 14 days (but up to 28 days) after uterine transposition surgery, highlighting the ability to maintain oncologic urgency and proceed with therapy in a timely fashion when successfully coordinated among multidisciplinary teams. After radiation therapy is completed, the uterus may be reimplanted in the pelvis for future pregnancy.

The optimal approach and reproductive outcomes of uterine transposition surgery are not yet fully established. One recent review article aimed to report the rates of uterine preservation, gonadal function, surgical complications, and pregnancy outcomes in patients who have undergone uterine transposition (84). At the time of its publication, there were 47 reported cases worldwide, with most patients undergoing uterine transposition for cervical cancer (55.6%) and rectal cancer (35.6%) (85). The most commonly reported surgical complication was cervical ischemia with vascular preservation of the uterine body; thus, surgeons should be aware of persevering descending vessels along the cervix (84). There have been no repositioning complications reported. All post-pubertal patients resumed ovarian hormonal and uterine function by means of regular menstruation after radiation treatment and repositioning. Four patients achieved spontaneous pregnancy and subsequently delivered healthy neonates via uncomplicated cesarean delivery at a gestational age between 36–38 weeks (85). Regarding obstetric risks for patients who have received uterine transposition surgery, data are too limited to draw any conclusions.

Several recent innovations have been introduced to advance the technique of uterine transposition. These include robotic versus conventional laparoscopic approaches (33), the use of human female cadaver models for both preclinical surgical training and radiotherapy dosimetry analysis (86, 87), and intraoperative assessment of uterine perfusion to ensure vascular integrity (88). Continued research is essential to identify the surgical approach that best balances technical complexity with effective transposition of the uterus to a location associated with minimal radiation exposure.

### 6.2 Research gaps and emerging directions for uterine surgery for fertility preservation

Despite growing interest in uterine surgery for fertility preservation, several key research gaps remain. These procedures have only been performed in a small number of cases, limiting robust conclusions about safety and efficacy. Long-term follow-up data on reproductive outcomes, graft function, and maternal-fetal health are lacking. Complication profiles, such as graft ischemia, surgical morbidity, and immunologic risks, are not yet fully characterized. Moreover, there is no standardized approach regarding surgical technique, timing relative to cancer treatment, or patient selection. Access to these procedures is also limited by high costs, lack of insurance coverage, and complex ethical, legal, and policy considerations that further hinder widespread implementation.

In contrast to uterine transposition, uterine transplantation (UTx) is a more established surgical approach that may offer a unique fertility preservation option for women who face uterine loss or dysfunction due to cancer treatment. UTx has demonstrated increasing success, with over 40 live births reported worldwide following both living and deceased donor transplants (89, 90). In the context of cancer, UTx is particularly relevant for survivors of gynecologic malignancies, such as cervical or endometrial cancer, or those requiring hysterectomy or pelvic radiation, where other fertility-preserving measures may not suffice (91, 92). While UTx has been successfully performed in patients with a history of hysterectomy for early-stage cervical cancer, there is a lack of clinical guidelines on patient selection, timing post-treatment, and graft surveillance specific to cancer survivors (93). The need for short term immunosuppressive therapy raises theoretical concerns about cancer recurrence, particularly in hormone-sensitive or immunologically driven cancers (94, 95). Nonetheless, early case reports and feasibility studies suggest that with rigorous screening and multidisciplinary oversight, UTx could become a viable fertility-restoring option for more cancer survivors in the future.

## 7 Cervical surgeries for fertility preservation

### 7.1 Radical trachelectomy

Radical trachelectomy is a fertility-preserving surgical option for patients with early-stage cervical cancer who wish to retain reproductive potential. This procedure is considered in reproductive-age individuals with stage IA1 disease exhibiting lymphovascular space invasion (LVSI), stage IA2–IB1 tumors less than 2 cm in diameter, and no evidence of nodal metastasis or deep stromal invasion. Select patients with tumors measuring 2–4 cm may also be candidates following neoadjuvant chemotherapy (96).

First described in 1987, radical trachelectomy involves removal of the cervix, vaginal margins, and parametrial tissue (96). Surgical approaches include vaginal, abdominal, and minimally invasive techniques (laparoscopic or robotic), each with distinct fertility and oncologic outcomes. One systematic review from 2020 found that vaginal radical trachelectomy was the most commonly performed (58%), followed by abdominal (37%) and laparoscopic (4.7%) approaches. It also demonstrated lower rates of preterm delivery. However, longer follow-up in this cohort may contribute to the observed fertility outcomes (97). Reported pregnancy rates among women attempting conception after radical hysterectomy varied widely in the literature, ranging from 25% to 95%, with most estimates exceeding 40% (98–102). In contrast, pregnancy rates with alternate approaches are low; abdominal trachelectomy has a reported 10% pregnancy rate, and both laparoscopic and robotic trachelectomy have a reported 9% pregnancy rate (97).

### 7.2 Simple trachelectomy and cold knife conization

**Figure 4:**
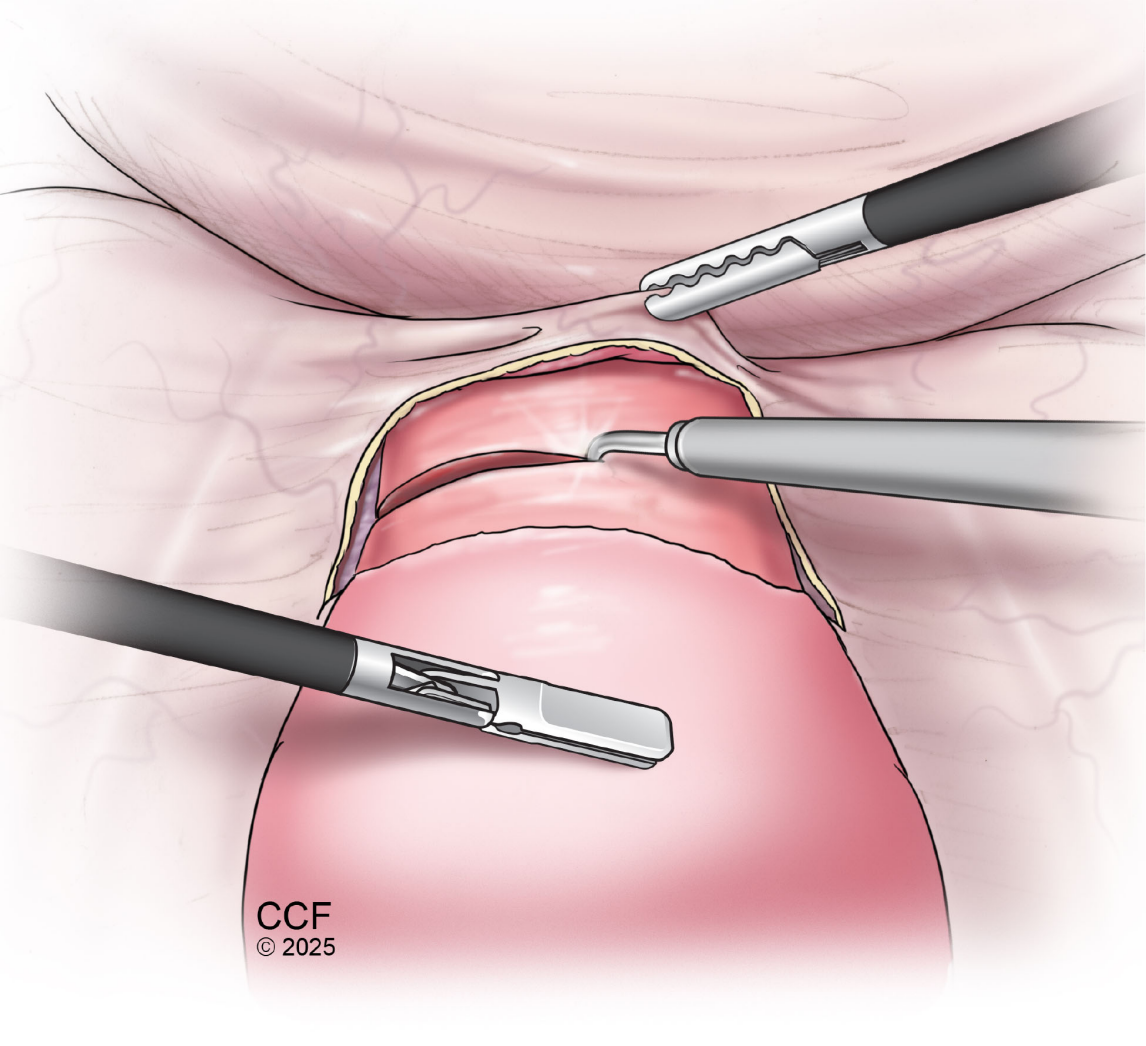
Laparoscopic simple trachelectomy for uterine preservation. Illustration of laparoscopic simple trachelectomy, demonstrating resection of the cervix while preserving the uterine corpus and adnexa. The procedure includes transection of the uterosacral and cardinal ligaments, identification and protection of the ureters, and division of the cervix at the level of the internal os. The vaginal cuff is subsequently closed, maintaining uterine integrity and allowing for preservation of uterine endocrine and reproductive potential in appropriately selected patients.

Simple trachelectomy is defined as removal of the cervix and possible removal of a small portion of the upper vagina (**Figure 4**). In comparison to radical trachelectomy, parametrial tissue is not removed. For patients with favorable pathology (those with FIGO 2009 stage IA2–IB1 squamous or adenocarcinoma, tumor size <2 cm, depth of invasion <10 mm, and negative pelvic nodes), the risk of parametrial involvement is <1%. In this cohort, simple trachelectomy or cold knife conization (CKC) may be considered definitive therapy (103). A recent international multicenter FERTISS study found that patients undergoing non-radical fertility-sparing surgery had significantly higher pregnancy rates compared to those receiving radical procedures, although LBR were similar between the two groups. Approximately half of the 733 patients attempted conception, with 22.6% achieving pregnancy. Pregnancy rates were 63.2% for non-radical surgeries (e.g., conization or simple trachelectomy) versus 25.7% for radical trachelectomy (p < 0.001). Most pregnancies (>89%) were spontaneous. While LBR did not differ, preterm birth (<38 weeks) was more frequent after radical trachelectomy. Notably, regular cervicometry and prophylactic management improved live birth outcomes (104).

## 8 Discussion

Surgical approaches to fertility preservation offer vital options for cancer patients seeking to maintain reproductive potential without compromising oncologic safety. Procedures such as ovarian transposition, ovarian tissue cryopreservation and transplantation, uterine-sparing surgeries, and fertility-preserving cervical surgeries represent an expanding toolkit for individualized care. As survival rates improve and fertility becomes a central quality-of-life consideration, these surgical strategies provide critical alternatives for patients who may not be candidates for standard cryopreservation techniques. Continued refinement of surgical techniques, alongside multidisciplinary collaboration and long-term outcome tracking, is essential to optimizing both oncologic and reproductive outcomes in this unique patient population.

### 8.1 Multidisciplinary approach and individualized care

Oncofertility is a rapidly evolving field that demands a multidisciplinary, patient-centered approach. Timely risk assessment, patient-centered counseling, and streamlined access to fertility preservation interventions are critical to avoid delays in cancer therapy and reduce decisional regret. Specifically, counseling should begin at diagnosis, with risk stratification for gonadotoxicity. If the risk of POI and infertility exceeds 50%, fertility preservation surgery should be offered and ideally completed prior to chemotherapy or radiation (105). Post-treatment assessment of endocrine and reproductive function is also recommended. Multidisciplinary oncofertility care is now considered the standard of care, with evidence suggesting improved patient satisfaction and long-term outcomes (105).

### 8.2 Implications for practice and future directions

The integration of fertility preservation into standard oncologic care represents a paradigm shift in survivorship planning. While ovarian transposition and fertility-preserving cervical surgeries are well-established, emerging techniques such as OTC and OTT, as well as uterine transposition or transplantation warrant further investigation. Ongoing research is needed to elucidate mechanisms of ischemia-related follicle loss, optimize graft revascularization, and better define the risk of malignant cell reintroduction. In addition, current studies of uterine transposition are limited by a lack of procedural standardization and long-term reproductive and endocrine outcome data.

The vast majority of studies across all surgical fertility preservation methods lack insight into technique variability, patient selection, and challenges posed to patients by both access and equity barriers. Longitudinal studies are needed to assess reproductive outcomes, safety profiles, and quality-of-life metrics following these interventions. Future research should also explore innovations in surgical technique, intraoperative imaging, and tissue engineering to enhance fertility preservation outcomes. Additionally, expanding access to fertility preservation services, particularly in underserved populations, remains a critical goal.

### 8.3 Conclusion

As cancer survival rates improve, reproductive health must be prioritized alongside oncologic outcomes. The integration of fertility preservation into standard oncologic care is imperative and requires a coordinated, multidisciplinary team. As the field advances, continued collaboration across specialties will be vital to ensure that fertility preservation becomes a standard, equitable component of comprehensive cancer care.
